# EGFR‐TKI plus antiangiogenic agent combination therapy may reduce the incidence of EGFR‐TKI–associated interstitial lung disease in Asian

**DOI:** 10.1002/kjm2.12584

**Published:** 2022-08-26

**Authors:** Tai‐Huang Lee, Hsiao‐Ling Chen, Ming‐Ju Tsai, Chih‐Jen Yang

**Affiliations:** ^1^ Department of Internal Medicine Kaohsiung Municipal Ta‐Tung Hospital Kaohsiung Taiwan; ^2^ Department of Pharmacy Kaohsiung Municipal Ta‐Tung Hospital Kaohsiung Taiwan; ^3^ Division of Pulmonary and Critical Care Medicine, Department of Internal Medicine Kaohsiung Medical University Hospital Kaohsiung Taiwan; ^4^ School of Post‐Baccalaureate Medicine College of Medicine, Kaohsiung Medical University Kaohsiung Taiwan

Dear Editor:

The most serious adverse drug reaction experienced by patients receiving epidermal growth factor receptor (EGFR) tyrosine kinase inhibitors (TKIs) is interstitial lung disease (ILD), which has an incidence of 1.6%–4.3% and a mortality rate of 13%–50% among Asian populations.^1^ However, the mechanism through which EGFR‐TKIs induce lung injury remains unknown. EGFR‐TKIs prevent the phosphorylation of EGFR, disrupting the regeneration and proliferation of cells necessary to repair the injured epithelium. EGFR‐TKIs might upregulate interleukin‐6 in cancer cells, inducing interstitial pneumonitis. Chronic inflammatory conditions in pulmonary tissues might also contribute to the occurrence of EGFR‐TKI–induced lung injury. Vascular endothelial growth factor (VEGF) interacts with VEGF receptor (VEGFR)–1, VEGFR–2, and VEGFR–3, and VEGF, platelet‐derived growth factor (PDGF), and fibroblast growth factor (FGF) have all been implicated in the pathogenesis of lung fibrosis. Therefore, antiangiogenic agents targeting VEGF may ameliorate lung fibrosis. Nintedanib, an antiangiogenic agent that inhibits PDGF receptor (PDGFR)α and β, FGF receptor (FGFR)1–3, VEGFR1–3, and transforming growth factor‐beta (TGF‐β), has been approved by the US Food and Drug Administration (FDA) for use as a standard agent in the treatment of idiopathic lung fibrosis.^2^ The antiangiogenic agents bevacizumab and ramucirumab were approved by the FDA for use in combination with the EGFR‐TKI erlotinib in patients with advanced nonsmall cell lung cancer who harbor susceptible *EGFR* mutations after large‐scale clinical trials found that combination therapy significantly prolongs progression‐free survival (PFS).^3^, ^4^, ^5^

Whether therapy combining an antiangiogenic agent with an EGFR‐TKI is able to reduce the incidence of ILD remains uncertain due to the rarity of EGFR‐TKI–induced ILD. To elucidate the ability of antiangiogenic agents to protect against EGFR‐TKI–induced ILD, we performed a comprehensive literature search of the PubMed, EMBASE, and Clinical-Trials.gov databases using the keywords “nonsmall‐cell lung cancer,” “EGFR Mutation,” “EGFRTKI,” “antiangiogenic agents,” “Interstitial lung disease (ILD),” and “randomized controlled trial.” Data from identified studies were assessed for quality using a risk of bias tool. Meta‐analysis was conducted using the Mantel–Haenszel method in Revman software to compare the risk ratio (RR) of ILD occurrence between EGFR‐TKI monotherapy and EGFR‐TKI plus antiangiogenic agent combination therapy. Heterogeneity was evaluated by the Chi‐square test and the I‐squared test. A total of 1314 patients from eight randomized controlled trials were included in our meta‐analysis. EGFR‐TKI plus antiangiogenetic agent combination therapy was associated with a significantly reduced risk of ILD incidence compared with EGFR‐TKI monotherapy (RR = 0.46, 95% confidence interval 0.25–0.82; Figure 1).

**FIGURE 1:**
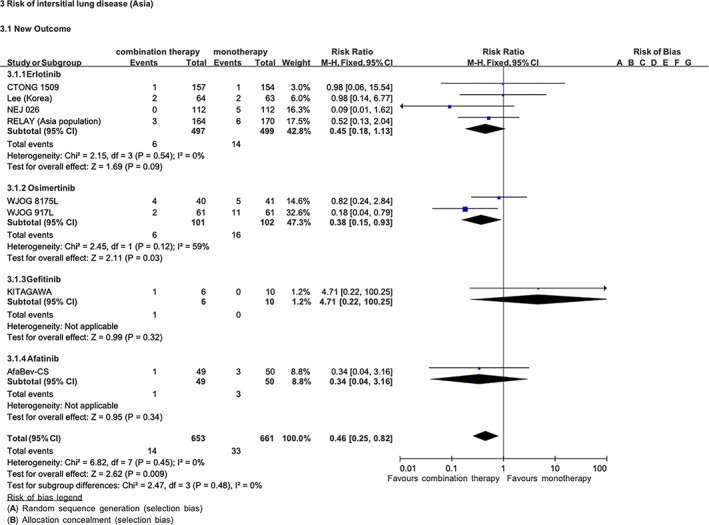
A total of 1314 patients from eight randomized controlled trials were included in our meta‐analysis. *The two most recently updated trials of combination therapy, one conducted by lee et al. (Korea) and the AfaBev‐CS trial (Japan), were reported at the 2022 American Society of Clinical Oncology (ASCO) meeting held from June 4 to June 8, 2022

In lung cancer, adding an antiangiogenic agent to EGFR‐TKI therapy has both advantages and disadvantages. Antiangiogenic agents may induce mild adverse reactions, such as epistaxis, hypertension, proteinuria, and bleeding; however, combination therapy with an antiangiogenic agent and an EGFR‐TKI appears to have clinical benefits. In addition to significantly prolonged PFS compared with EGFR‐TKI monotherapy, we identified, for the first time, the potential that combination therapy may reduce ILD incidence among Asian populations.

## CONFLICT OF INTEREST

All authors declare no conflict of interest.

## References

[1] Ohmori T , Yamaoka T , Ando K , Kusumoto S , Kishino Y , Manabe R , et al. Molecular and clinical features of EGFR‐TKI‐associated lung injury. Int J Mol Sci. 2021;22(2):792. 33466795 10.3390/ijms22020792 PMC7829873

[2] Wollin L , Wex E , Pautsch A , Schnapp G , Hostettler KE , Stowasser S , et al. Mode of action of nintedanib in the treatment of idiopathic pulmonary fibrosis. Eur Respir J. 2015;45(5):1434–45. 25745043 10.1183/09031936.00174914 PMC4416110

[3] Saito H , Fukuhara T , Furuya N , Watanabe K , Sugawara S , Iwasawa S , et al. Erlotinib plus bevacizumab versus erlotinib alone in patients with EGFR‐positive advanced non‐squamous non‐small‐cell lung cancer (NEJ026): interim analysis of an open‐label, randomised, multicentre, phase 3 trial. Lancet Oncol. 2019;20(5):625–35. 30975627 10.1016/S1470-2045(19)30035-X

[4] Seto T , Kato T , Nishio M , Goto K , Atagi S , Hosomi Y , et al. Erlotinib alone or with bevacizumab as first‐line therapy in patients with advanced non‐squamous non‐small‐cell lung cancer harbouring EGFR mutations (JO25567): an open‐label, randomised, multicentre, phase 2 study. Lancet Oncol. 2014;15(11):1236–44. 25175099 10.1016/S1470-2045(14)70381-X

[5] Zhou Q , Xu CR , Cheng Y , Liu YP , Chen GY , Cui JW , et al. Bevacizumab plus erlotinib in Chinese patients with untreated, EGFR‐mutated, advanced NSCLC (ARTEMIS‐CTONG1509): a multicenter phase 3 study. Cancer Cell. 2021;39(9):1279–91.e3. 34388377 10.1016/j.ccell.2021.07.005

